# Drivers of the composition and diversity of carabid functional traits in UK coniferous plantations

**DOI:** 10.1016/j.foreco.2015.10.008

**Published:** 2016-01-01

**Authors:** Rebecca Spake, Nadia Barsoum, Adrian C. Newton, C. Patrick Doncaster

**Affiliations:** a Centre for Biological Sciences, Institute for Life Sciences Building 85, University of Southampton, Southampton SO17 1BJ, UK; b Centre for Ecosystems, Society and Biosecurity, Forest Research, Alice Holt Lodge, Farnham, Surrey GU10 4LH, UK; c Centre for Conservation Ecology and Environmental Science, Faculty of Science and Technology, Bournemouth University, Talbot Campus, Fern Barrow, Poole, Dorset BH12 5BB, UK

**Keywords:** Carabids, Functional diversity, Functional traits, Plants, Plantation forest, Trait-based approach

## Abstract

Functional diversity (FD) is increasingly used as a metric to evaluate the impact of forest management strategies on ecosystem functioning. Management interventions that aim to maximise FD require knowledge of multiple environmental drivers of FD, which have not been studied to date in temperate coniferous production forests. We quantified the relative importance of abiotic (forest management) and biotic (ground vegetation community) drivers of carabid FD and trait distribution in 44 coniferous plantation forest stands across the UK. Carabid FD declined with canopy cover and carabid body length correlated negatively with the percentage of open semi-natural area surrounding a plot. We conclude that forest management could enhance carabid FD through initiatives that emulate natural disturbance regimes through gap creation. We found that neither functional nor taxonomic metrics of vegetation diversity correlated with carabid FD, suggesting that restoration of plant communities, a major goal of forest restoration efforts, will not necessarily enhance carabid FD in coniferous plantations.

## Highlights

• We investigated drivers of carabid functional trait composition and diversity (FD).
• We used data from 44 coniferous plantations across the UK.
• Carabid FD declined with canopy cover.
• Carabid body length decreased with the percentage of open habitat surrounding a stand.
• Neither functional nor taxonomic metrics of ground vegetation diversity indicated carabid FD.

## 1 Introduction

Research on the impacts of environmental change on invertebrate biodiversity has traditionally adopted a taxonomic approach by focusing on the composition and diversity of particular groups of species in a community (Fountain-Jones et al., 2015). This approach has limited scope for generalisations, especially when comparing different regions with different species pools (McGill et al., 2006). A recent shift towards consideration of functional traits has focused attention on the morphological, anatomical, biochemical, physiological or phenological traits of a species that influence its ability to acquire resources, disperse, reproduce and persist in the environment (Violle et al., 2007, Pavoine and Bonsall, 2011). Functional-trait analysis offers several advantages over taxonomic composition analysis for both conceptual and management purposes (McGill et al., 2006, Kleyer et al., 2012). It facilitates an understanding of the mechanisms that underlie both community responses to environmental change and ecosystem functioning (Diaz et al., 2007, Lavorel et al., 2008, Laliberte et al., 2010, Bachand et al., 2014).

Quantitative measures have been developed that use multivariate methods to integrate multiple traits into a single continuous trait diversity index. These measures capture the value, range or distribution of functional traits in a community (hereafter ‘functional diversity’; FD; Hooper et al., 2005, Diaz et al., 2007). Unlike traditional taxonomic measures of species richness or diversity, FD presupposes a mechanistic link between diversity and the ecological phenomena in question (Cadotte et al., 2011), and it has become apparent that FD is more directly relevant to ecosystem functioning than taxonomic diversity (TD; Hooper et al., 2005, Díaz and Cabido, 2001, Vandewalle et al., 2010). It is thought that predator assemblages exhibiting high diversity in functional traits are likely to have high levels of complementarity in traits associated with natural enemy capture and consumption (Petchey and Gaston, 2002). Conversely, assemblages with low FD may be more likely to exhibit niche overlap, increasing interference competition and limited potential for biological control (Woodcock et al., 2010). A quantitative review by Gagic et al. (2015) revealed that functional trait-based indices of animal diversity consistently provided greater explanatory power than species richness or abundance in predicting various ecosystem functions including pollination and pest control. It follows that estimates of invertebrate FD may provide surrogate measures of such services (Woodcock et al., 2014).

Considering that species differ in their response to environmental factors and effects on ecosystem functioning, it is important to understand the impacts of environmental changes on biotic communities. In this study, we investigate the diversity and distribution of functional traits for carabid beetles and ground layer plants, in relation to environmental variation imposed by forest management in coniferous production forests in the UK. Carabid beetles are a diverse and abundant group of insects ubiquitous to most terrestrial ecosystems (Thiele, 1977), where they contribute to ecosystem functioning through predation of other invertebrates and granivory of plant seeds (Lang et al., 1999, Kotze et al., 2011). In forest ecosystems, carabid beetles are important natural enemies of insect pests (Alalouni et al., 2013) and contribute to nutrient cycling (Loreau, 1995, Prather et al., 2013). Carabids have experienced general declines in diversity and abundance across the UK, but have remained relatively stable in forest and hedgerow habitats (Brooks et al., 2012), presenting opportunities for forest management to increase their value as carabid biodiversity refuges at the landscape level (Brooks et al., 2012).

Management of plantation forests requires an understanding of the environmental drivers affecting FD across taxonomic groups in order to sustain the multifunctional roles of these forests. It has been suggested this appreciation will require unravelling complex biotic interactions (Gilman et al., 2010, Brooks et al., 2012). Trait-based extensions to multi-taxa assessments are consequently being advocated as a means to further our understanding of community assembly following disturbance (Moretti and Legg, 2009, Aubin et al., 2013, Bachand et al., 2014). In temperate forests, most vascular plant species occupy the ground layer, where they form the trophic and structural template for a diversity of invertebrate communities (Sabatini et al., 2014). Plant functional traits mediate interactions with the physical environment, which suggests that data on plant and invertebrate traits may reveal more than species compositional data about the relationships between these taxa and their interactions with the environment (Moretti and Legg, 2009). Trait-based multi-taxa approaches therefore permit analyses of the relative influences of abiotic drivers (e.g. forest management) and biotic drivers (e.g. the plant community) on responses by animal communities to disturbance events (Pakeman and Stockan, 2014).

In this study, we investigate the diversity and distribution of carabid and ground layer plant functional traits, in relation to environmental variation imposed by forest management. We use data from the Biodiversity Assessment Project, which has previously been analysed only from a taxonomic perspective (Jukes et al., 2001, Humphrey et al., 2003). The BAP found that carabid species diversity declined with increasing canopy cover and soil organic matter content, and that the abundance of forest specialist carabid species increased with stand age whilst non-woodland species declined (Jukes et al., 2001). The influence of forest management on carabid FD has received less attention. Aubin et al. (2013) studied the FD of several taxa, including carabids, in boreal plantation forests, in relation to the single environmental variable of stand age. Pakeman and Stockan (2014) considered multiple abiotic and biotic drivers of carabid in arable fields, pasture and moorland. Our study is the first to analyse responses of carabid functional trait composition and diversity in relation to environmental drivers in planted coniferous production forests. This type of forest comprises around a half (52%) of total UK forest area (Forestry Commission, 2012). Our objective is to determine the processes driving carabid community dynamics in coniferous forest plantations. We use chronosequence data from 44 conifer plantations distributed widely across the UK (Humphrey et al., 2003). Specifically, we set out to: (i) compare the relative importance of a number of abiotic and biotic drivers of carabid FD in plantation forests; (ii) test whether meaningful correlations exist between carabid FD and taxonomic and functional metrics of ground vegetation diversity (species richness, Shannon-Wiener, Simpson diversity and Rao’s quadratic entropy); and (iii) identify a combination of functional traits in ground layer plant and carabid species that are most sensitive to forest management and which could potentially be used to characterise priority groups for conservation action.

## 2 Materials and methods

### 2.1 The Biodiversity Assessment Project

All analyses used the dataset of the UK Forestry Commission’s Biodiversity Assessment Project (BAP) which ran from 1995 to 1999. Here we summarise relevant features of sampling design (Humphrey et al., 2003).

#### 2.1.1 Study location and design

**Fig. 1:**
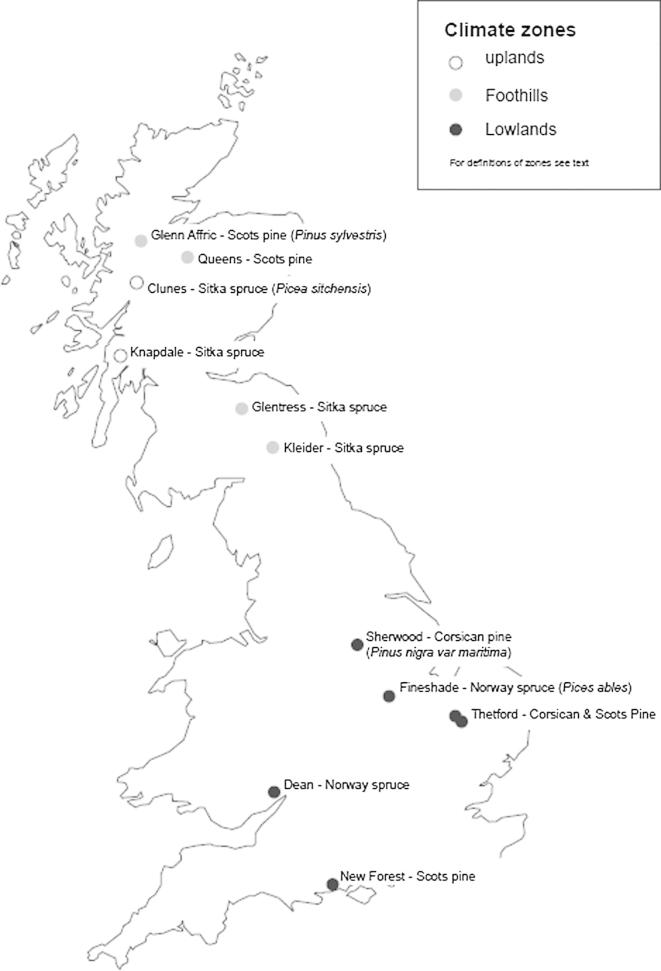
Location of the 12 coniferous sites assessed by the Biodiversity Assessment Project; 44 plots representing four stand age classes across chronosequences (see text) were sampled over a 4-year period.

Conifer plantation stands at 12 sites across the UK were selected for study (Fig. 1; Table 1). These comprised four prominent commercial crop types grown in the UK: Sitka spruce (*Picea sitchensis* L. Bong. Carr.), Scots pine (*Pinus sylvestris* L.), Corsican pine (*Pinus nigra* var. maritime L.), and Norway spruce (*Picea abies* L. Karst.).

At the 12 sites, 1-ha permanent sample plots were established in four forest stands, reflecting four growth stages of a typical commercial timber crop rotation. Humphrey et al. (2003) provide a full description of these structure classes. Each site comprised of plots dominated by a single crop type. The chronosequence stages used included (i) a pre-thicket restock stage, crop height 2–4 m, age 8–10 years, incomplete canopy closure; (ii) a mid-rotation stage – crop height 10–20 m, age 20–30 years, canopy closure, no understorey; (iii) a mature stage – crop height 20–25 m, age 50–80 years, canopy closure, some development of understorey layers; and (iv) an over-mature stage (beyond economic maturity and acquiring some of the ecological characteristics of natural old-growth forests *sensu* Oliver, 1981) – crop height > 25 m, age 60–250 years, canopy break-up, well-developed understorey layers, accumulation of deadwood. A randomised-block design was used to assign the four growth stages to each site. In practice, the overmature age class was not present at four of the sites, resulting in a working total of 44 plots. Furthermore, sites were not equally distributed across climatic zones in the BAP project, meaning that not all combinations of tree species and bioclimatic zones were represented, leading to these factors being confounded.

#### 2.1.2 Ground vegetation sampling

Two 10 × 10-m quadrats were arranged diagonally across the centre of each 50 × 50-m quarter of the 1-ha plot, giving eight quadrats in total per plot. The composition of ground vegetation (vascular plants) was assessed visually using the DOMIN cover-abundance scale *sensu* Dahl and Hadač (1941), within eight 2 × 2-m quadrats nested within the 10 × 10-m quadrats. To aggregate quadrat values to the plot level, each quadrat DOMIN score was converted to an average percentage, the percentages were summed and divided by eight. This value was then assigned the appropriate score on the DOMIN scale.

#### 2.1.3 Ground beetle sampling

Ground beetles were sampled by pitfall trapping. Five traps were positioned 10 m apart on a north–south transect through the centre of each 1-ha plot and trapping was carried out over a 20-week period from May to September for two consecutive years (Table 1) and emptied at fortnightly intervals. Pitfall trap catches were pooled to the plot level for analysis. Carabid identification was based on Forsythe (1987) and Lindroth (1974). Due to difficulties with taxonomy, *Pterostichus rhaeticus* Heer (Luff, 1990) was recorded as *Pterostichus nigrita* (Paykull). Similarly, *Asaphidion curtum* Heyden and *Asaphidiom stierlini* Heyden (Speight et al., 1986) were not separated from *Asaphidion flavipes* (L.).

#### 2.1.4 Environmental data

Environmental variables that have previously been observed to affect carabid diversity and composition in plantation forests (Jukes et al., 2001), were selected to interpret differences in carabid FD and trait composition between stands (Table 2). Bioclimatic zones were uplands, foothills and lowlands, delineated by annual rainfall totals of: >1500 mm (uplands); 800–1500 mm (foothills); and <800 mm (lowlands), following the Forestry Commission’s Ecological Site Classification (ESC – Pyatt et al., 2001). Four vegetation strata S1 to S4 were defined, covering field, shrub, lower canopy and upper canopy layers. Percentage cover of vegetation within each vertical stratum was described to the nearest 5%. A general measure of canopy cover for carabids was given by the percentage cover of vegetation in S3: lower canopy, due to its correlation with leaf area index and consequent influence over light levels at the forest floor (Ferris et al., 2000, Jukes et al., 2001).

### 2.2 Trait selection and calculation of functional diversity

#### 2.2.1 Trait selection criteria and trait databases

Functional traits were selected *a priori* using published literature (Table 3), within the constraints of data availability. We selected traits thought to mediate direct responses of vegetation and beetle communities to the environmental changes imposed by forest management. These were traits related to morphology, reproduction, dispersal and resource use (Bachand et al., 2014). We also selected traits thought likely to capture indirect effects of the forest cycle on beetle communities through bottom-up control by plants. The ‘structural heterogeneity hypothesis’ posits that bottom-up control of invertebrate communities is exerted through the physical structure of the vegetation, by affecting microhabitat specialisation, hunting efficiency and vulnerability of invertebrates to their predators (Brose, 2003). We selected traits thought to underpin these mechanisms (Table 3).

#### 2.2.2 Calculation of functional diversity

Rao’s quadratic entropy (FD*_Q_*; Rao, 1982, Botta-Dukát, 2005) is a multi-trait FD metric that describes the variation of species trait composition within the community. It sums pairwise distances between species in a community weighted by their relative abundances. We calculated FD*_Q_* for each plot as: ${\text{FD}}_{Q}=\sum_{i=1}^{N}\sum_{j=1}^{N}d_{\mathrm{ij}}p_{i}p_{j}$ where *N* is the number of species in a plot, *d_ij_* is the difference in trait values between the *i*th and *j*th species; *p_i_* and *p_j_* are the proportions of the *i*th and *j*th species, calculated as number of individuals per species relative to the total number of individuals in the community. Functional distances between species were calculated using Gower’s distance metric, which allows for a mixture of continuous, ordinal, and categorical variables, and accommodates missing trait values (Laliberte and Legendre, 2010, Sonnier et al., 2014). Continuous trait data were scaled by range to assign equal weighting amongst traits (Botta-Dukát, 2005). FD*_Q_* possesses all of the necessary properties of a FD index including its representation of the range of character values present and its ability to be relatively unaffected when a minor species with an extreme character value decreases in abundance (Botta-Dukát, 2005). It has widespread use and has been shown to successfully identify habitat filtering patterns (de Bello et al., 2009, Moretti and Legg, 2009, Aubin et al., 2013).

### 2.3 Statistical analyses

#### 2.3.1 Abiotic and biotic drivers of carabid functional diversity

All analyses were computed in R 3.00 software (R Core Team, 2013). Linear mixed models were used to quantify the effects of environmental variables on carabid FD*_Q_*. Explanatory variables included forest stage, tree species, bioclimatic zone, % open ground and cover by vegetation strata, soil type, and vegetative FD*_Q_* (Table 2). Missing combinations of tree species across climatic zones precluded cross-factoring of these variables; we therefore ran these partially crossed factors as a single combined factor ‘treesp_clim’ with as many levels as existing combinations of these factors. We fitted our mixed model following the protocol of Zuur et al. (2013). In our global model, site was incorporated as a random factor since plots within a given location were expected to be similar and should not be considered independent. We fitted appropriate dependency structures *a priori* that allowed for different slopes of the relationships between carabid FD and covariates across sites where appropriate. All possible additive models were constructed using maximum likelihood methods in package MuMIn (Barton, 2013), to allow model comparisons based on Akaike’s Information Criterion with small-sample correction (AICc; Burnham and Anderson, 2004). We applied full model averaging (Lukacs et al., 2009) across all plausible models – those with Akaike weights summing to at least 0.95 – because the minimum adequate model as selected by AICc was not strongly weighted (Symonds and Moussalli, 2011). The goodness of fit of each plausible model was estimated by calculating the marginal *R*^2^ following Nakagawa and Schielzeth (2013). Relationships between carabid and FD and covariates were graphed using coefficients from the minimum adequate model refitted using restricted maximum likelihood.

Key requirements of indicators include their ease of application and ability to be applied with confidence in novel contexts (McGeoch, 1998). This means that they should correlate with biodiversity independently of other factors. Therefore, we explored simple bivariate relationships to investigate whether taxonomic or functional metrics of vegetation diversity (vegFD*_Q_*) were better predictors of carabid FD. We tested for a meaningful correlation of carabid FD*_Q_* with measures of vegetation diversity based on Pearson’s *r*, or Spearman’s rho when variables violated parametric assumptions. For taxonomic measures of vegetation diversity, we used species richness, and the Shannon-Wiener and Simpsons diversity indices which account for species’ relative abundances.

#### 2.3.2 Functional trait associations with environmental variables

Prior to analysis, abundance values were log-transformed to reduce the effect of dominant species (Ribera et al., 2001). We applied fourth-corner analysis (Dray and Legendre, 2008, Dray et al., 2014) to measure and test directly the correlation between the variations in carabid and vegetation species traits and the environmental variables using the ‘ade4’ package (Dray and Dufour, 2007). Two permutation tests were applied to determine correlation as recommended by Dray and Legendre (2008). Firstly, we tested the null hypothesis that species assemblages are randomly attributed to plots, irrespective of plot environmental conditions, using 4999 permutations of plot vectors (Model 2 of Dray and Legendre, 2008). Secondly, we tested the null hypothesis that species traits have no influence on species composition of samples with fixed environmental conditions, by permuting species vectors (Model 4 of Dray and Legendre, 2008). From these models, we chose the larger of the two *P* values as the most conservative approach (ter Braak et al., 2012). To account for multiple testing of environmental variables and multiple traits, we adjusted *P* values to account for false discovery rates. We report results both with and without this correction, because correction can increase the likelihood of a type II error rate (Rothman, 1990).

## 3 Results

### 3.1 Environmental drivers of carabid functional diversity

**Fig. 2:**
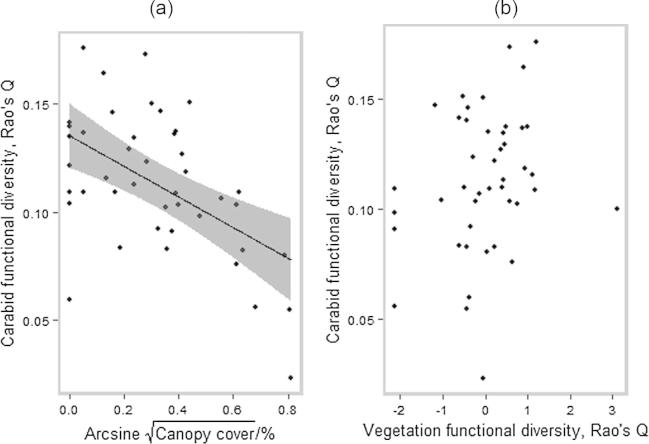
Carabid functional diversity variation with (a) canopy cover, and (b) vegetation functional diversity. Vegetation diversity has been centred and scaled. Regressions used coefficients of the minimum adequate model based on AICc and REML estimation. Grey shading shows 95% prediction intervals based on uncertainty in fixed effects only.

Model selection and multimodel inference suggested that the most important variable controlling carabid FD*_Q_* was cover within the S3 stratum (hereafter ‘canopy cover’; Table 4, Table 5), which negatively affected carabid FD (Fig. 2). Canopy cover featured in all plausible models. vegFD*_Q_*, % open, and treesp_clim did not appear consistently across these models (Table 4), contributing to their relatively low importance values of 0.40–0.45 (Table 5). The model containing canopy cover as the only fixed effect had a marginal *R*^2^ value of 0.35 (Table 4). The variables S1, S2 and stage had barely any importance in explaining variation in carabid FD across plots (Table 5) and did not appear in models with ΔAICc < 2, i.e. models with substantial support (Burnham and Anderson, 2004). Full model averaging revealed canopy cover to be the only variable to significantly explain carabid FD (Table 5).

Our analysis of simple bivariate relationships between carabid FD and vegetation diversity metrics found no detectable correlation with carabid FD*_Q_* for either functional or taxonomic metrics of vegetation diversity (*r* values of 0.24–0.26; Figs. 2 and S1).

### 3.2 Environmental drivers of species trait distributions

Fourth corner analysis detected correlations of environmental variables with vegetation and carabid functional traits. The correlation matrix of classes of vegetation traits by classes of environmental variables detected four significant correlations. Plots with high percentage canopy cover associated with vegetation communities that had low tolerance to light (*P *< 0.01), and that dispersed by means other than wind dispersal (*P *< 0.01); Scot’s pine plots were dominated by woody species (*P *< 0.01), and Norway spruce stands by phanerophytic plant species (*P *= 0.01). The correlation matrix of classes of carabid traits by classes of environmental variables detected four significant correlations. Carabid communities that overwinter as adults dominated in plots with high percentage canopy cover (*P *= 0.02); carabid body length correlated negatively with the percentage of open semi-natural area surrounding a plot (*P *= 0.04); omnivorous carabid communities dominated in Norway spruce plots (*P *= 0.04); carabid communities that favoured open habitats correlated negatively with Sitka spruce plots (*P *= 0.04). None of these eight associations remained detectable after correcting for multiple comparisons using the false discovery rate, except the association of Scot’s pine plots with woody species (*P* < 0.01).

## 4 Discussion

### 4.1 Environmental drivers of functional diversity and trait distribution

Our study reveals that canopy cover is the most important driver of carabid FD in UK coniferous production forests, tending to drive down carabid FD (Fig. 2). Other studies, including a taxonomic analysis of this dataset, have found that carabid TD declines with canopy cover or stand age (Spake et al., in press, Jukes et al., 2001, Vanbergen et al., 2005, Gibb and Cunningham, 2010). Open-habitat specialists are expected to decrease with increasing canopy cover, and to re-establish as plantations approach, or pass, maturity when the canopy becomes less continuous through tree mortality and/removal through management. This response may be mediated by gap size and proximity to open habitat (Jukes et al., 2001, Toigo et al., 2013). Open-habitat specialists have functional traits that differ from those of forest specialists, for example tending to be winged and smaller in size, reflecting higher dispersal abilities (Jelaska and Durbesic, 2009). Our observation of declining overall carabid FD with canopy cover can be attributed to an absence of open-habitat species in plots with high canopy cover. This was observed in the fourth corner analysis, with the negative association between open-habitat species and Sitka spruce plots prior to correction for multiple comparisons; it was also observed in the taxonomic community analysis of the same dataset by Jukes et al. (2001). Sitka spruce plots exhibited a larger mean and range in canopy cover than the other tree species, which each had similar mean values of canopy cover. Previous studies have found a positive correlation of carabid body size with canopy cover (Gibb and Cunningham, 2010) and percentage forest cover in the surrounding landscape (Vandewalle et al., 2010). Our results support the latter finding, with fourth corner analysis revealing a negative correlation between body size and the percentage of open habitat surrounding landscape. We found high cover to be associated with species that overwinter as adults. Such species will complete their larval stages during the summer, and may therefore select habitat with high cover to reduce the risk of larval desiccation.

Ground vegetation was not an important driver of carabid FD, with a relatively low importance value of 0.45 (Fig. 2; Table 5). Previous taxonomic analysis of the dataset found that vegetation diversity (Shannon-Wiener) was not important in determining carabid species richness or diversity (Jukes et al., 2001). This finding is contrary to Moretti and Legg’s (2009) assertion that relationships between taxa are more likely to be revealed when using a functional, rather than a taxonomic approach, as functional traits represent interactions between organisms and their environment. Indeed, Pakeman and Stockan (2014) demonstrated positive correlations between measures of vegetation and carabid FD in agricultural setting in the UK. In their study and ours, traits used to calculate vegetation and carabid FD were related to morphology, reproduction, dispersal and resource use. For plants, these were mostly broad morphological traits, suggesting that ground vegetation FD is likely to reflect the degree of structural heterogeneity. Brose (2003) outlines three hypotheses that may underpin a direct relationship between assemblages of invertebrate and structural aspects of the vegetation. These are: (i) microhabitat specialisation, in which vertical or horizontal zonation is higher in architecturally complex plant communities that offer microsites for oviposition, hibernation and shelter; (ii) hunting efficiency, in which vegetation structure changes the efficiency of different hunting strategies causing large predators to be more efficient in sparse vegetation; and (iii) enemy-free space, in which vegetation structure affects the vulnerability of prey species that have more chance of escaping from natural enemies in dense vegetation. It is also possible that higher vegetation FD supports increasing numbers of specialised invertebrate consumers (Murdoch et al., 1972), which through cascade effects can encourage predator assemblages with greater divergence in traits related to consumption (Hunter and Price, 1992).

### 4.2 Management implications

#### 4.2.1 Maintaining high carabid FD in production forests

The capacity for forests to sustain diverse and stable carabid communities suggests that forest management could aim to maximise their value as carabid refuges in agricultural landscapes (Brooks et al., 2012). The decline of carabid FD with cover observed in our study supports the implementation of silvicultural treatments that emulate natural disturbance regimes through canopy gap creation, such as close-to-nature forestry. Gap-based management has been included in proposals for managing forests across the world (Muscolo et al., 2014). Canopy gap creation will also benefit ground layer plant communities in UK coniferous plantations. Plant species with high levels of tolerance to light could be lost in productive landscapes with high canopy cover, as shown by the negative correlation between light tolerance and cover in this study. That being said, the value of gaps in terms of the communities they contain depends on a host of factors including gap size (Latty et al., 2006), spatio-temporal distribution (Marthews et al., 2008) and shape (Garbarino et al., 2012), which were not considered in this study and must be addressed by gap-based management regimes.

The restoration of plant communities has become a major goal of forest conservation efforts such as restoration initiatives, with the assumption that the conditions that lead to more diverse vegetation will also lead to a restoration of insect communities (Babin-Fenske and Anand, 2010). Our observation of no relationship between vegetation diversity (either taxonomic or functional; Figs. 2 and S1) and carabid FD suggests that management strategies that aim to promote a functionally diverse understorey shrub layer will not necessarily enhance carabid FD in coniferous plantations.

#### 4.2.2 Utility of vegetation FD as an indicator of carabid functional diversity

Indicators of biodiversity are required for judging the success of management regimes intended to sustain biodiversity (Lindenmayer et al., 2000), and for prioritisation of protected areas (Larsen et al., 2009), as a surrogate for costly and time-consuming inventories of total biodiversity. In forests, ground layer plants are amongst the most commonly studied taxa when identifying potential surrogates for other taxa, typically invertebrates (Wolters et al., 2006). A large literature has emerged quantifying the extent of cross-taxon congruence for a range of taxonomic groups, spatial locations and scales. Westgate et al. (2014) performed a global meta-analysis of these studies and revealed a high variance in cross-taxon congruence. Their analyses suggest that there are few circumstances in which pairs of taxa will be consistent surrogates for each other across a range of metrics, locations and spatial scales (Westgate et al., 2014). Given that a key requirement of indicators is their ability to be applied with confidence in novel contexts (McGeoch, 1998), this lack of consistency casts doubt on the generalizability of taxonomic surrogates in ecology and conservation planning (Westgate et al., 2014). The authors emphasise the need for novel approaches to the study of cross-taxon congruence and posit that functional metrics of biodiversity could be considered as potential means to improve surrogacy. Here we show that cross-taxon congruence is thought to have any of several causes: (i) random coincidence; (ii) interactions between taxa, (iii) similar responses to common environmental variables, and (iv) similar responses to different, but spatially covariant, environmental variables (Gaston, 1996, Wolters et al., 2006). Functional metrics are likely to perform better as surrogates than taxonomic metrics, as they incorporate species’ morphological, anatomical, biochemical, physiological or phenological traits associated with a species’ ability to acquire resources, disperse, reproduce and persist in the environment (Violle et al., 2007), and therefore reflect interactions with the environment and between species (Moretti and Legg, 2009, Gillison et al., 2013). Our results show that the incorporation of functional traits commonly used to assess community responses to the environment does not necessarily improve cross-taxon congruence, particularly in circumstances where other environmental variables (in our case, canopy cover) have a high importance in determining biodiversity.

## 5 Conclusion

Invertebrate functional diversity (FD) is directly relevant to ecosystem functions including pollination and pest control and is therefore being increasingly used as a metric to evaluate the impact of forest management strategies. Despite this, the majority of research on the impacts forestry on invertebrate biodiversity has focussed on drivers of taxonomic diversity. Our investigation of the drivers of carabid FD amongst temperate coniferous production forest stands across the UK which vary in environmental conditions as imposed by forest management including crop species, stand developmental stage and variation in canopy cover has shown that canopy cover is an important driver of carabid FD, with increasing cover tending to drive down FD. Contrary to previous studies, we found that ground vegetation diversity is not an important determinant of carabid FD, and its performance as a surrogate is not improved when functional metrics of ground vegetation are used over taxonomic metrics. This suggests that conservation or management efforts that restore diverse plant communities will not necessary benefit carabid communities, but those that emulate natural disturbance through canopy gap creation will.

## Figures and Tables

**Table 1:** Metrics of sample sites used in assessment of carabid community composition, 1995–1997.

	Site	UK grid reference	Forest category	Tree species	Sampling years
1	Glen Affric, Highland	NH 317363	Foothills	Scots pine	1996–1997
2	Strathspey, Highland	NH 853084	Foothills	Scots pine	1996–1997
3	Thetford, Norfolk	TL 833901	Lowland	Scots pine	1995–1996
4	New Forest, Hants.	SU 254064	Lowland	Scots pine	1995–1997^a^
5	Knapdale, Argyll	NR 814907	Upland	Sitka spruce	1995–1996
6	Clunes, Highland	NN 257938	Upland	Sitka spruce	1995–1996
7	Forest of Dean, Gloucs.	SO 608130	Lowland	Norway spruce	1995–1996
8	Fineshade, Northants.	SP 982978	Lowland	Norway spruce	1995–1996
9	Kielder, Northumberland	NY 715860	Foothills	Sitka spruce	1996–1997
10	Glentress, borders	NN 257938	Foothills	Sitka spruce	1996–1997
11	Thetford, Norfolk	TL 815760	Lowland	Corsican pine	1996–1997
12	Sherwood, Notts.	SK 606621	Lowland	Corsican pine	1996–1997

a Plots within site #4 were sampled in 1995–1996, except for the over-mature plot which was sampled in 1996–1997.

**Table 2:** Environmental variables and vegetation functional diversity used to explain variation in carabid functional diversity.

Variable code	Description	Range or levels
Stage	Chronosequence stage	Pre-thicket (Pre)/mid-rotation (Mid)/mature (Mat)/overmature (Omat)
Treesp	Crop type	Scots pine/Norway spruce/Corsican pine/Sitka spruce
Clim	Bioclimatic zone	Uplands/foothills/lowlands
% open	Percentage cover of open semi-natural area including farmland, grassland and heathland within 1-km radius of plot	0–50; continuous
S1	Field, 10 cm – 1.9 m high	0–75; continuous
S2	Shrub, 2–5 m high	0–40; continuous
S3	Lower canopy, 5.1–15 m high	0–55; continuous
S4	Upper canopy, 15.1–20 m high	0–30; continuous
Soil	Soil type	Podzolic/peaty gleys/surface water gleys/acid brown earths/calcareous brown earths and clays
vegFD*_Q_*	Ground vegetation diversity calculated as Rao’s quadratic entropy	0.000–0.144; continuous

**Table 3:** Plant and beetle traits used to calculate functional diversity metrics.

Trait (code)	Type/unit	Trait range or category	Trait data source(s)
*Plants*			
Height (height)	Continuous/mm	10–600	(3), (8)
Leaf area (lf.area)	Continuous/mm		(3), (8)
Raunkier life form (life)	Ordinal	Geophyte/therophyte/hemicryptophyte/chamaephyte/phanerophyte	(3)
Ellenberg shade tolerance (light)	Ordinal	1–9 (1 = plant in deep shade; 9 = plant in full light)	(3)
Stem woodiness (woody)	Categorical	Woody/non-woody	(3)
Wind dispersal (wind)	Categorical	Yes/no	(4)

*Ground beetles*			
Body length (length)	Continuous/mm	2.95–30	(2)
Adult feeding guild (diet)	Categorical	Collembola specialist/generalist predator/phytophagous/omnivorous	(2), (5), (10)
Hind-wing morphology (wing)	Categorical	Macropterous/dimorphic/apterous or brachypterous	(2), (5), (7)
Activity pattern (active)	Categorical	Diurnal/nocturnal	(9), (10)
Adult habitat affinity	Categorical	Forest/open/generalist	(1), (6), (12)
Breeding season	Categorical	Spring/summer/autumn or winter	(1), (10), (12)
Overwinter type	Categorical	Adult only/larvae or adult	(10), (11)

(1) Desender et al. (1994); (2) Edgar and Purvis (2014); (3) Fitter and Peat (1994); (4) Hintze et al. (2013); (5) Homburg et al. (2014); (6) Jukes et al. (2001); (7) Luff (2007); (8) Kleyer et al. (2008); (9) Pakeman and Stockan (2014); (10) Ribera et al. (2001); (11) Stork (1990); and (12) Thiele (1977).

**Table 4:** Most parsimonious linear mixed models of carabid functional diversity as measured using Rao’s quadratic entropy (FD*_Q_*). Only models with substantial support are shown, with ΔAICc < 2, ranked by AICc weight (Burnham and Anderson, 2004).

Model	Fixed explanatory variables included in model^a^	df	ΔAICc	AICc weight	Marginal *R*^2^
1	S3 + vegFD*_Q_*	6	0.00	0.11	0.37
2	S3	5	0.20	0.10	0.35
3	S3 + clim	10	0.44	0.09	0.30
4	S3 + % open + treesp + vegFD*_Q_*	12	1.00	0.07	0.61
5	S3 + % open + treesp	11	1.25	0.06	0.58
6	S3 + % open	6	1.27	0.06	0.32
7	S3 + % open + vegFD*_Q_*	7	1.42	0.05	0.36
8	S3 + treesp + vegFD*_Q_*	11	1.61	0.05	0.58
Null		4	15.25	0.00	0.00
Global	vegFD*_Q_* + S3 + % open + stage + soil + treesp	17	15.44	0.00	0.69

a See Table 2 for variable codes.

**Table 5:** Full model-averaged parameter estimates and importance values for models of carabid functional diversity whose cumulative Akaike weight summed to 0.95, calculated by multiplying the estimates for individual models which contain parameters by their weights. Relative importance is the sum of the AICc weights across these models.

Explanatory variable	Parameter estimate	Standard error	*P*	Importance value
Intercept	0.126	0.013	<0.001	
S3	−0.074	0.017	<0.001	1.00
vegFD*_Q_*	0.111	0.154	0.711	0.45
Sitka spruce_foothill	0.000	0.008	0.976	
Sitka spruce_uplands	0.006	0.010	0.563
Corsican pine_lowland	0.018	0.021	0.403
Norway spruce_lowland	0.009	0.013	0.486
Scots pine_lowland	0.018	0.022	0.404
Perc	0.011	0.017	0.528	0.41
S1	0.002	0.008	0.850	0.18
S2	−0.001	0.009	0.891	0.15
Stage_Pre	−0.001	0.005	0.798	
Stage_Mid	−0.001	0.004	0.852
Stage_Overmature	−0.001	0.004	0.838
