# Supplementary material for: Drivers of the composition and diversity of carabid functional traits in UK coniferous plantations
Source: For Ecol Manage. 2016 Jan 1;359:300–8. doi: 10.1016/j.foreco.2015.10.008 (PMC4705869; doi:10.1016/j.foreco.2015.10.008)
Supplement: Supplementary data 1 [file mmc1.docx]

## Supporting Information

### Appendix S1: Correlations between taxonomic ad functional metrics of vegetation diversity with carabid functional diversity


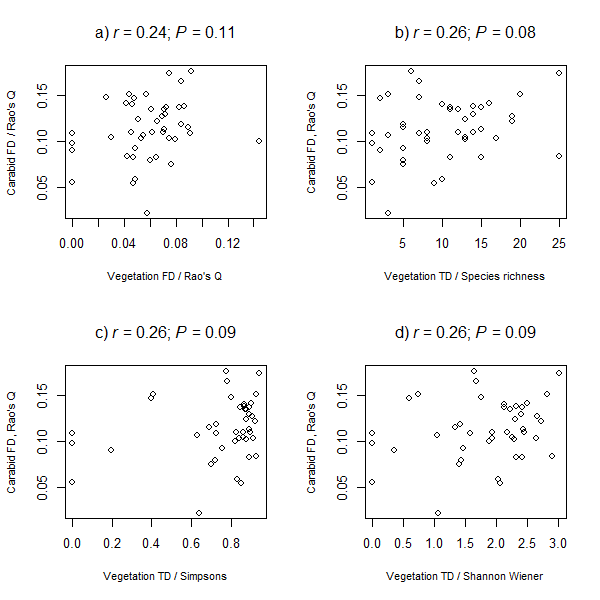


**Figure S1.**  Bivariate relationships between carabid functional diversity (FD) and a) vegetation FD; b) vegetation species richness; c) vegetation Simpsons diversity; and d) vegetation Shannon diversity.
